# Gross anatomy of the gluteal and posterior thigh muscles in koalas based on their innervations

**DOI:** 10.1371/journal.pone.0261805

**Published:** 2022-09-14

**Authors:** Sayaka Tojima, Hidaka Anetai, Kaito Koike, Saori Anetai, Kounosuke Tokita, Chris Leigh, Jaliya Kumaratilake

**Affiliations:** 1 Hakubi Center, Kyoto University, Kyoto, Japan; 2 The Kyoto University Museum, Kyoto University, Kyoto, Japan; 3 Department of Anatomy and Cell Biology, Graduate School of Medicine, Osaka City University, Osaka, Japan; 4 Department of Anatomy and Life Structures, Juntendo University School of Medicine, Tokyo, Japan; 5 School of Physical therapy, Faculty of Health and Medical Care, Saitama Medical University, Saitama, Japan; 6 Department of Global Agricultural Sciences, Graduate School of Agricultural and Life Sciences, The University of Tokyo, Tokyo, Japan; 7 Faculty of Health and Medical Sciences, Adelaide Medical School, The University of Adelaide, Adelaide, Australia; 8 Division of Research and Innovation, The University of Adelaide, Adelaide, Australia; 9 Biological Anthropology and Comparative Anatomy Research Unit, Adelaide Medical School, The University of Adelaide, Adelaide, Australia; New York Institute of Technology, UNITED STATES

## Abstract

Morphological and functional comparison of convergently-evolved traits in marsupials and eutherians is an important aspect of studying adaptive divergence in mammals. However, the anatomy of marsupials has been particularly difficult to evaluate for multiple reasons. First, previous studies on marsupial anatomy are often uniformly old and non-exhaustive. Second, muscle identification was historically based on muscle attachment sites, but attachment sites have since been declared insufficient for muscle identification due to extensive interspecific variation. For example, different names have been used for muscles that are now thought to be equivalent among several different species, which causes confusion. Therefore, descriptions of marsupial muscles have been inconsistent among previous studies, and their anatomical knowledge itself needs updating. In this study, the koala was selected as the representative marsupial, in part because koala locomotion may comprise primate (eutherian)-like and marsupial-like mechanics, making it an interesting phylogenetic group for studying adaptive divergence in mammals. Gross dissection of the lower limb muscles (the gluteal and the posterior thigh regions) was performed to permit precise muscle identification. We first resolved discrepancies among previous studies by identifying muscles according to their innervation; this recent, more reliable technique is based on the ontogenetic origin of the muscle, and it allows for comparison with other taxa (i.e., eutherians). We compared our findings with those of other marsupials and arboreal primates and identified traits common to both arboreal primates and marsupials as well as muscle morphological features unique to koalas.

## Introduction

Marsupials are a group of mammals that diverged from eutherians, a group which includes us humans, about 130 million years ago [1]. Several cases of convergent evolution between marsupials and placentals are known, whereby morphological similarities were acquired through adaptation to similar environments, such as seen in moles and marsupial moles, wolves and Tasmanian tigers, flying squirrels and sugar gliders, etc. In relation to the similarities in morphology, it is known that and eutherians and marsupials converge in locomotion patterns. For example, the locomotion of the quadrupedal and arboreal primates (eutherian) is characterized by diagonal-sequence gaits, which is thought to have evolved as a result of the necessity to move on thin and flexible branches. However, the non-primate arboreal marsupial, woolly opossum (*Caluromys philander*), has been shown to have foot morphology similar to that of primates and to exhibit similar locomotion [2, 3]. Not only one-to-one correspondence, but complex convergent evolution has occurred between species as well. According to a previous study, the locomotion of koalas contains both primate-like and marsupial-like locomotion patterns [4]. Clarifying how convergently-evolved traits are similar in function is important for an understanding of adaptive divergence in mammals, and thus, a comparative morphological study between marsupials and eutherians is essential. In the case of a koala, questions such as the following arise: do koala limb muscles have many primate characteristics? Or do they acquire a similar locomotor style through a muscular morphology that is completely different from that of primates? In order to make comparisons with the muscular morphology of phylogenetically-distant arboreal primates, it is necessary to identify the muscles by homologous criteria.

In general, the key to comparing anatomical morphology among species is the assurance of muscle homology (the method of how muscle identification is performed). In classical anatomy, muscle identification is performed based on the positional relationship between muscles and their attachment sites. Of course, this information is indispensable for estimating the range of motion and function of muscles. However, it is also known that attachment sites in the limbs of tetrapods vary greatly among species to allow for a variety of locomotion [5]. In other words, although information regarding muscle attachment site is useful for estimating muscle function, it is not sufficient by itself as a criterion for muscle identification. Therefore, muscle identification based on innervation of the muscles has emerged as an alternative to conventional muscle identification methods. This method is based on the nerve-muscle interactions during embryonic development [6–8] and reflects the developmental derivation of the muscles. Since the 1960’s, developmental biology has acknowledged important interactions between muscle derivations and innervation. It is important to consider the innervation to muscles as it is essential to muscle function for two reasons: nerve–muscle interaction during the developmental process and specificity between nerves and muscles [6–8]. Developmental biological studies have shown that normal muscular development and differentiation are strongly dependent on neural activities [9–11]. In early stage of musculogenesis, the passage of nerves strongly contributes to the separation of the muscle mass [5]. The separated muscle mass is then further subdivided to form individual muscles, and since there is specificity between the nerves and muscles [11], information about innervation is essential to establish the identity of the muscle. In fact, descriptions of anatomical muscle morphology based on this innervation have been accumulated in eutherians [12–24], and inter-species comparisons have been performed (e.g. Emura [25]).

On the contrary, the number of studies investigating gross anatomy in marsupials are much fewer than in eutherians. Additionally, most of the studies are classical, as they were conducted in the 1800–1900’s. In previous studies on marsupial anatomy, even the muscle names are not universal and muscles with similar attachment sites are sometimes given multiple names that differ between species, leading to confusion (quadrutus femoris [26]; caudo-femoralis [27]; ischiofemoralis [28]). Several classical studies have previously described the myology of koalas around the turn of the twentieth century [26, 29–31]. Along with other marsupial anatomy, even basic information about the muscular morphology in koalas was not fully clarified. Macalister [29] dissected the entire body of a female koala, but not all muscles were observed, and information on their attachments was insufficient. Sonntag [30] compared the anatomy of the wombat (*Phascolomys mitchelli*), koala, and phalanger (*Phalanger orientalis*); however, detailed information on each species was limited. Similarly, not all the attachment sites were described in the study by Macalister [29]. Elftman [31] compared pelvic region muscles among multiple marsupial species and produced drawings of the hip and thigh muscles. However, information about the attachments in dissected samples was not provided. Only Young [26] has summarized the attachments of koala muscles thoroughly. Another problem with the previous studies is the inconsistency in the description of muscles. For instance, Macalister [29] and Young [26] described that *m*. *gluteus medius* (GM) was bilaminar, while Sonntag [30] reported that there were no laminations. Thus, several reports of information regarding muscle attachments remain debatable and hence, require updating and reappraisal. The hind limb muscles of marsupials, especially the gluteal regions where multiple muscles overlap in layers, have been considered difficult to compare to those of eutherian mammals [28]. These inconsistencies occurred because the previous studies identified muscles only by their attachment sites. Nevertheless, even relatively recently reported anatomical descriptions of muscle identification have been compared only by muscle attachment site (e.g. Warburton [32]). As different species have different muscle types, muscle identification only by attachment sites may make comparative dissection difficult within marsupials as well as with that of eutherians.

Therefore, muscle identification must be based on a more solid homology. Very recently, comparative dissections based on innervation have been performed in the marsupial koala and the eutherian cat [33]; the focus of the study was the innervation of the shoulder girdle muscle group, and it also clarified the homological relationship of the cervico-thoracic trunk muscles. This is a groundbreaking work that presents the first clear muscle homology between marsupials and eutherians.

In this study, we specifically focused on the gluteal and posterior thigh muscle groups in koalas, which have been suggested to exhibit a combined primate-marsupial locomotion pattern [4]. Gluteal and posterior thigh regions are related to hip and knee joints and thus, thought to exhibit morphology consistent with this locomotion pattern. In addition, these regions have been considered particularly difficult to identify (one-to-one comparison with eutherians) because of the multiple layers of muscles in these regions [28]. Therefore, the primary objective of this study is to reorganize and update the knowledge of muscular morphology in this complex but important region of the lower limb according to irrefutable criteria and eliminate discrepancies of previous findings. We are confident that we will be able to demonstrate the usefulness of muscle identification through innervation. Another objective of this study is to establish a basis for comparative anatomy of marsupials and eutherians by comparing the anatomical findings of a koala with those of arboreal primates, which have been suggested to have similar locomotion patterns.

## Material and methods

### Koala specimens

Five adult koala specimens (four males and one female) were used in this study (Table 1). All animals had been previously fixed and stored in 10% buffered formalin after having their thoracic and abdominal cavities dissected and digestive organs removed These specimens had previously been used for teaching comparative anatomy at the University of Adelaide, but their hind limbs were intact. The original weight of the samples is unknown because of the incision on the chest and abdomen; removal of the organs had been performed before the authors’ dissection. They were housed at the Discipline of Medicine, the University of Adelaide, South Australia. The animals were originally collected frozen from Cleland Wildlife Park under the Permit number K23749-12 from the Department of Environment, Water and Natural Resources, Government of South Australia. No animals were killed for this study.

**Table 1:** Koala specimens used in this study.

No.	Sex	Side
1	Male	Both sides
2	Female	Both sides
3	Male	Both sides
4	Male	Right side
5	Male	Right side

### Dissection

In this study, muscles of the gluteal region and posterior thigh and the nerves innervating them were dissected macroscopically in detail (Table 2) and identified based on the innervation (see Introduction for the history of muscle identification methods). Previous studies have reported that lower-limb morphology is more specialized than upper-limb morphology in marsupials [34]. It is also suggested that koala locomotion contains a mixture of both arboreal-primate-like and marsupial-like elements [4]. Therefore, we focused our dissection on the gluteal and posterior thigh muscles, which are thought to contribute to koala locomotion, have been in disagreement with previous studies, and require significant reorganization, in order to establish a foundation for comparative anatomical discussion.

**Table 2:** Observed koala muscles in this study.

**Gluteal muscles**					
	**Name**	**Abbreviation**	**Innervation**	**Origin**	**Insertion**
1	*M*. *gluteus superficialis*	Gsu	a branch from the sacral plexus (L8-S1)	Sacro-caudal region (S3-Ca3)	Femur shaft
2	*M*. *gluteus medius* (superficial part)	GMS	the superior guluteal nerve (L7-L8)	Sacro-caudal region (S1-S3), iliac crest	the great trochanter
	*M*. *gluteus medius* (deeper part)	GMD	the superior guluteal nerve (L7-L8)	sacro-iliac ligament	the great trochanter
3	*M*. *gluteus minimus*	Gmi	the superior guluteal nerve (L7-L8)	the ilium	the great trochanter
4	*M*. *piriformis*	PI	a branch from the sacral plexus (S1)	Sacral body (ventral side)	the great trochanter
5	*M*. *ischiofemoralis*	IF	a branch from the sacral plexus (S1)	The median sacral crest, sacrotuberous ligament, the ischial tuberosity	Femur shaft
**Posterior thigh muscles**					
	**Name**	**Abbreviation**	**Innervation**	**Origin**	**Insertion**
1	*M*. *biceps femoris*	BF	deep tibial nerve (DTN, a branch from the sacral plexus))	the ischial tuberosity	tibia
2	*M*. *semitendinosus*	ST	DTN	the ischial tuberosity	medial conjoint tendon with *M*.*gracilis*
3	*M*. *hamstring bundle*	HB	DTN	ST	BF
4	*M*. *semimembranosus*	SM	DTN	the ischial tuberosity	the tibia

## Results

We successfully identified five types of muscles in the gluteal region and four types of muscles in the posterior thigh, based on their innervations and attachments. In this section, we described each of the muscles exposed from the superficial to deep layers. There were no variations in the innervations and attachments by individual or sex.

**Fig 1:**
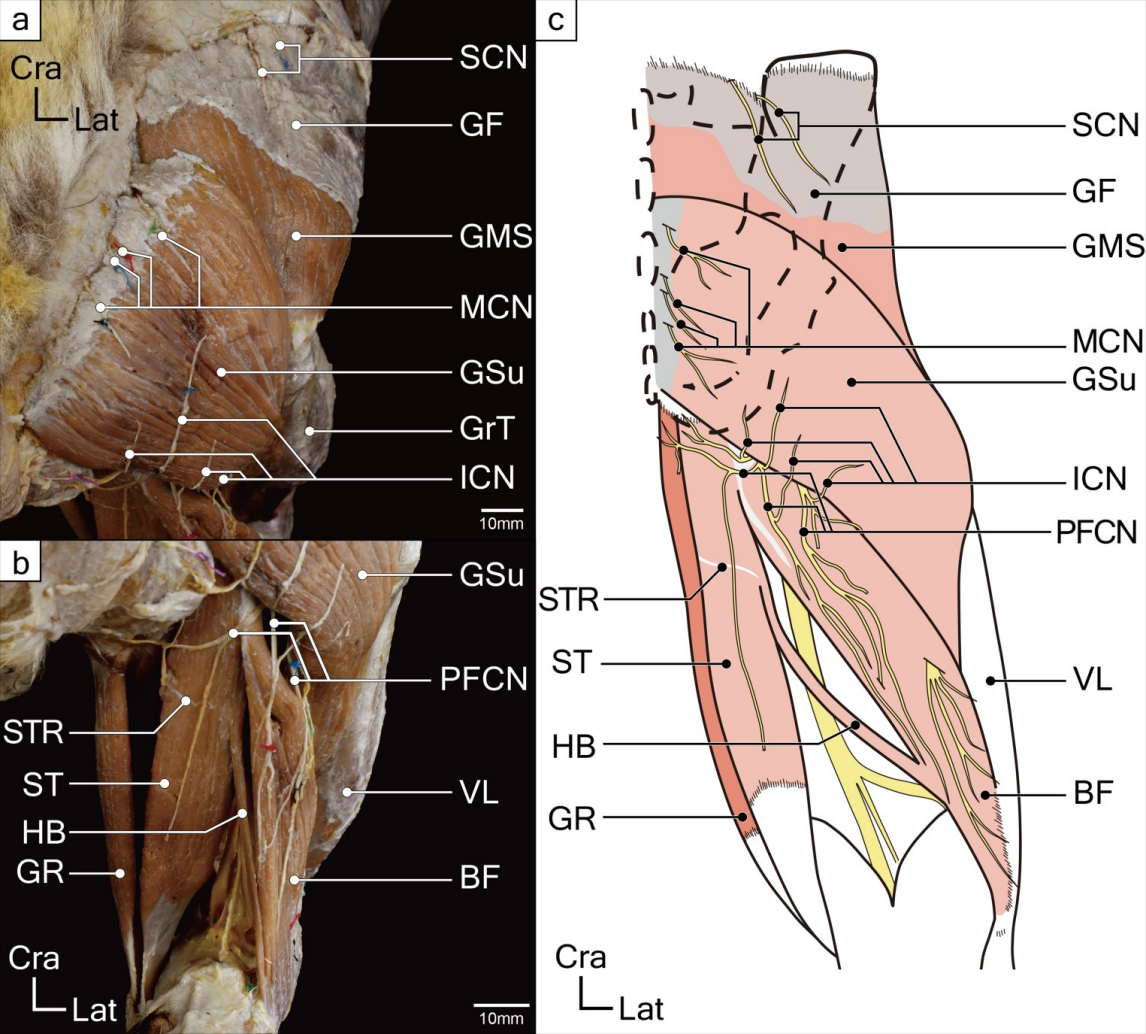
Superficial layer muscles of gluteal and posterior femoral regions in a koala right hind limb. (**a**) The superficial gluteal muscles, the gluteus superficialis (GSu) and superficial part of the gluteus medius (GMS), and some cutaneous nerves are shown. (**b**) The thigh flexors and some cutaneous nerves are shown. (**c**) Illustrated schematic drawing of the superficial layer muscles of gluteal and posterior femoral regions. Abbreviations: BF, biceps femoris; GF, gluteal fascia; GMS, superficial layer of the gluteus medius; GR, gracilis; GrT, greater trochanter; GSu, gluteus superficialis; HB, hamstring bundle; ICN, inferior cluneal nerve; MCN, middle cluneal nerve; PFCN, posterior femoral cutaneous nerve; SCN, superior cluneal nerve; ST, semitendinosus; STR, semitendinosus raphe; VL, vastus lateralis.

**Fig 2:**
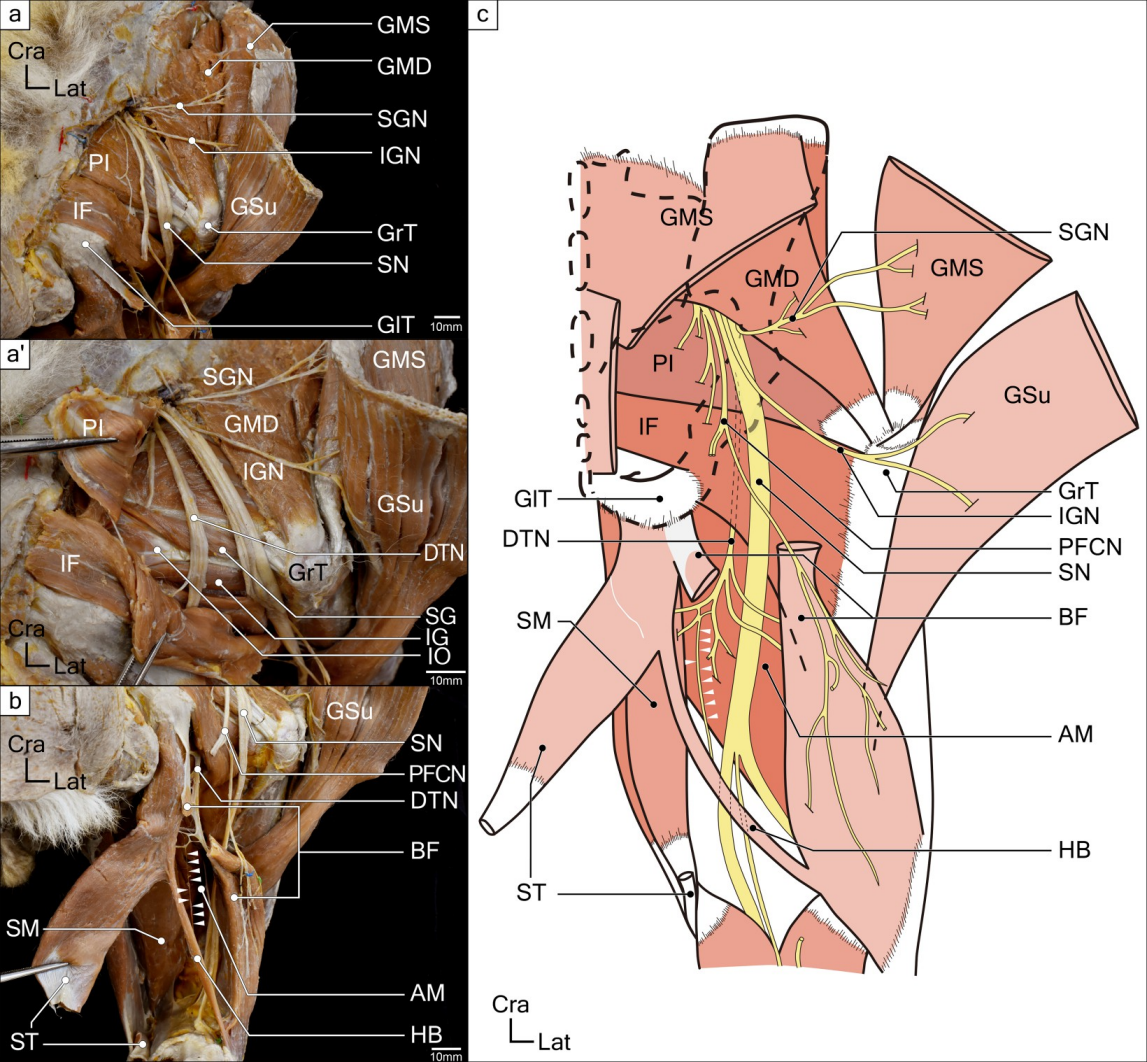
Deep layer muscles of gluteal and posterior femoral regions in a koala right hind-limb. (**a**) The deep gluteus muscles and several nerves including muscular branches are shown. (**a**) The deep tibial nerve (DTN) and deep gluteus muscles deep to the piriformis (PI) and ischiofemoralis (IF) are shown. (**b**) The innervating branches to the thigh flexors are shown. (**c**) Schematic drawing of deep gluteal muscles, thigh flexors, and innervating branches. White arrowheads indicate the innervating branch from the deep tibial nerve (DTN) to the hamstring bundle (HB). Abbreviations: AM, adductor magnus; BF, biceps femoris; DTN, deep tibial nerve; GlT, gluteal tuberosity; GMD, deep layer of the gluteus medius; GMS, superficial layer of the gluteus medius; GrT, greater trochanter; GSu, gluteus superficialis; HB, hamstring bundle; IF, ischiofemoralis; IG, inferior gemellus; IGN, inferior gluteal nerve; PFCN, posterior femoral cutaneous nerve; PI, piriformis; SG, superior gemellus; SGN, superior gluteal nerve; SM, semimembranosus; SN, sciatic nerve; ST, semitendinosus; VL, vastus lateralis.

**Fig 3:**
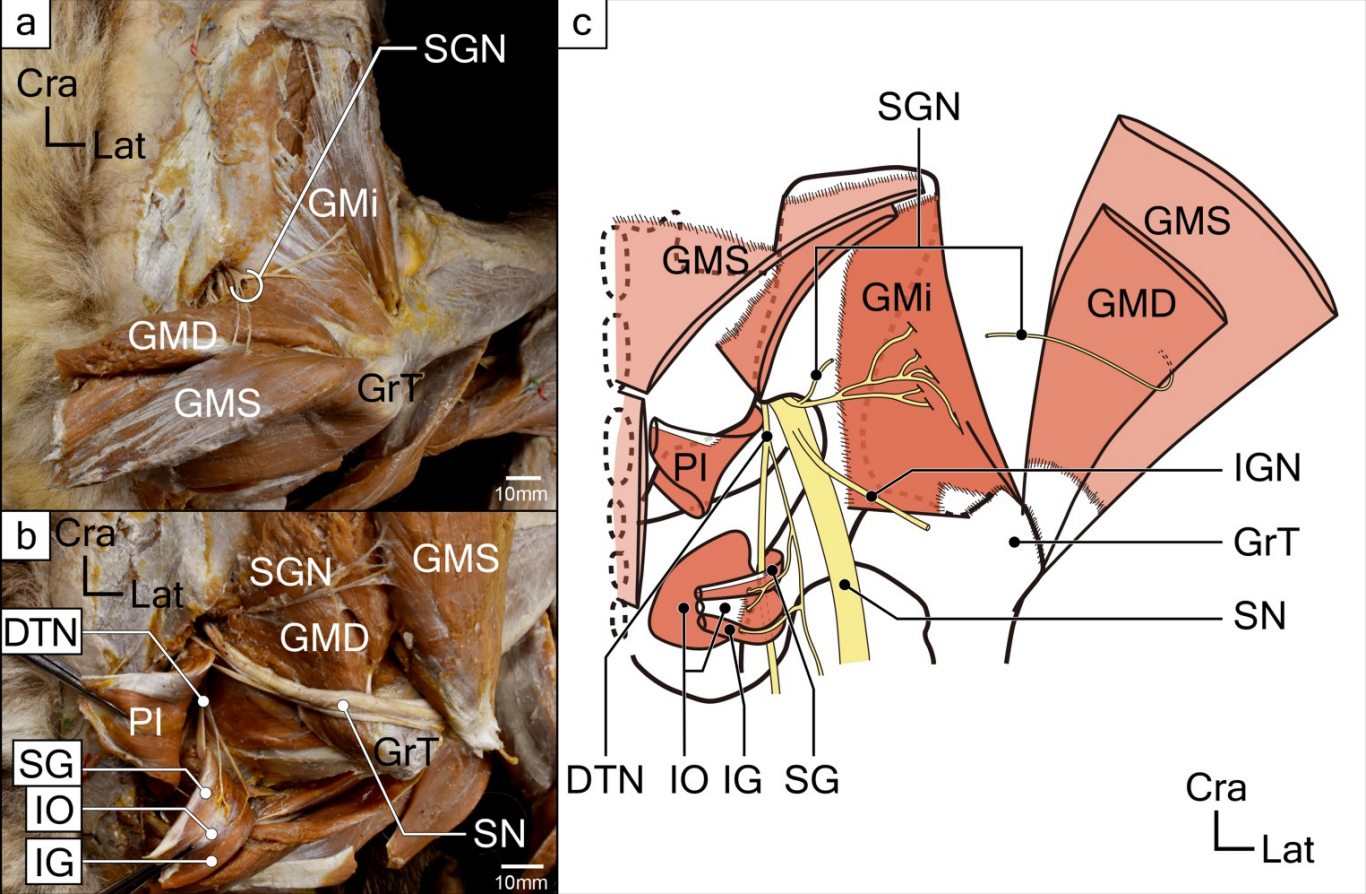
Deepest layer muscles of gluteus region and its innervations in koala right hip. (**a**) The superficial and deep layers of the gluteus medius (GMS and GMD), gluteus minimus (GMi), and the innervating branch from the superior gluteal nerve (SGN) are shown. (**b**) Gemellus and internal obturator muscles and supplying branch from the deep tibial nerve (DTN) are shown. (**c**) Schematic drawing of the deepest layer of gluteus muscles and its innervations. Abbreviations: DTN, deep tibial nerve; GlT, gluteal tuberosity; GMD, deep layer of the gluteus medius; GMi, gluteus minimus; GMS, superficial layer of the gluteus medius; GrT, greater trochanter; IG, inferior gemellus; IGN, inferior gluteal nerve; IO, internal obturator; PI, piriformis; SG, superior gemellus; SGN, superior gluteal nerve; SN, sciatic nerve.

**Fig 4:**
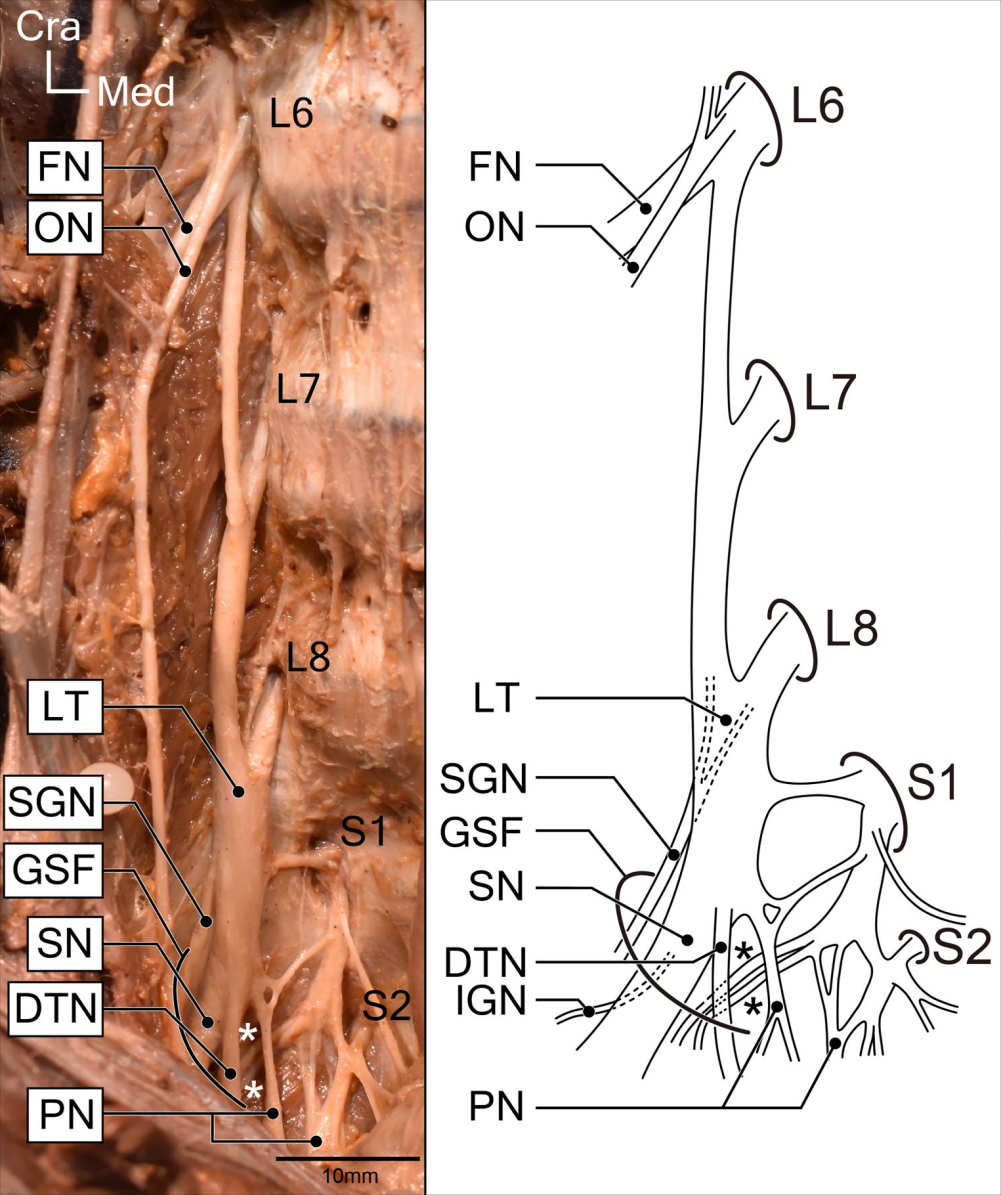
Right lumbosacral plexus in koalas. Photograph and illustrated corresponding schematic drawing. White and black asterisks indicate branches forming the posterior femoral cutaneous nerve and the innervating branch to the piriformis and ischiofemoralis after leaving the greater sciatic foramen. Abbreviations: DTN, deep tibial nerve; FN, femoral nerve; GSF, greater sciatic foramen; IGN, inferior gluteal nerve; LT, lumbosacral trunk; ON, obturator nerve; PN, pudendal nerve; SGN, superior gluteal nerve; SN, sciatic nerve.

All koalas dissected in this study possessed eight lumbar and three sacral vertebrae. The nerves innervating each muscle were tracked back to their roots and identified (Figs 1–4). The sacral plexus in koalas comprised the most caudal branch of the sixth lumbar nerve (L6) to the second sacral nerve (S2).

### Muscles in the gluteal region

#### 1. *M*. *gluteus superficialis* (GSu)

The GSu muscle was located in the most superficial layer eneath the superficial fascia. The muscle originated from the posterior aspect of the sacro-caudal region (from the third sacral vertebra to the third caudal vertebra) and was inserted onto the middle of the posterior surface of the femur shaft. The site of insertion extended approximately from 5 cm distal to the greater trochanter to 3 cm proximal to the distal end of the femur. There were no attachments to the ilium. These findings are consistent with the description of the gluteus maximus in several previous studies [26, 29, 30].

A branch from the sacral plexus (L8 to S1 nerves) entered the deep surface of the muscle. It was not a branch of the sciatic nerve, but possibly of the inferior gluteal nerve (Figs 1 and 2). Branches of the cutaneous nerves passed through the muscle near its origin (Fig 1).

#### 2. *Superficial* (GMS) and *deep* (GMD) *M*. *gluteus medius* (GM)

The GM muscle was located deeper than the GSu muscle. It originated from the posterior sides of the first to the third sacral vertebrae and the iliac crest and was inserted onto the lateral side of the greater trochanter. The muscular fascia separated the muscle into the GMS and GMD parts (Figs 2 and 3). The deep part originated along the shallow part of the posterior sacro-iliac ligament and was inserted onto the greater trochanter as a common tendon with the superficial component of the muscle.

The nerve that innervated this muscle originated from the L7 to L8 nerve roots, i.e., the superior gluteal nerve, entered the muscle from the deep surface and supplied both its GMD and GMS parts. Several branches of the cutaneous nerves supplying the skin on the superior part of the gluteal region passed through the cranial and medial parts of the GMS belly (Fig 1).

#### 3. *M*. *gluteus minimus* (GMi)

The GMi muscle, with a single belly, was located deeper than the GMD. It originated from the surface of the ilium and was inserted onto the greater trochanter. The nerve that supplied this muscle branched from the one that innervated the GM (the superior gluteal nerve). This nerve entered from the superficial aspect of the muscle belly.

#### 4. *M*. *piriformis* (PI)

The PI muscle was exposed after the transecting and reflecting of the GSu and GM muscles. It originated from the ventral side of the sacral body and was inserted onto the greater trochanter. The sciatic nerve (L6-S2) passed above this muscle, i.e., the suprapiriform foramen, and then descended (Figs 1–3). The nerve innervating this muscle branched from the sacral plexus (S1), close to the posterior femoral cutaneous nerve, and then entered the muscle belly from the superficial surface. No other muscle was observed to be homologous to the piriformis, except this muscle. Taking its attachments and innervations into account, it was identified as the piriformis muscle in koalas.

#### 5. *M*. *ischiofemoralis* (IF)

This muscle was located in the same layer as PI and was caudal to it. The origin was from the median sacral crest, sacrotuberous ligament, and the ischial tuberosity. The insertion was broad and expanded from the posterior edge of the greater trochanter to the shaft of the femur (i.e., just above the insertion of the GSu) (Fig 3).

The nerve innervating this muscle branched from the nerve supplying PI (S1), and then entered the muscle belly from the superficial surface at the superior proximal edge (Fig 3). This precluded finding a homologous muscle in other species. The attachment site and location of the IF observed in this study is consistent with the description given by Young [24] as quadrutus femoris but differs in attachment site and innervation from the quadrutus femolis muscle possessed by eutherians [35]. First, the muscle observed in koalas is attached to the sacrum and sacrospinous ligament with a more medial origin and a broader insertion more distal to the trochanteric fossa. In addition, the koala muscle only has a branch of the nerve that enters from the posterior (superficial) surface of the trochanteric fossa and does not have an anterior (deep) branch like the quadratus femoris muscle in other eutherian mammals [35]. Finally, this muscle is located in a different layer (more superficially) than the superior and posterior gemellus muscles and the internal obturator muscle. Considering its origin, insertion, innervation, and stratification, we concluded that it was not the same as the so-called quadratus femoris muscle. Considering the site of attachment, we think it is appropriate to call it *m*. *ischiofemolis*.

Furthermore, the nerves innervating *m*. *semitendinosus* (ST), *m*. *semimembranosus* (SM), *m*. *biceps femoris* (BF), and *m*. *adductor magnus* (AM) descended on the deep surface of this muscle, and we conveniently referred to it as the “deep tibial nerve (DTN).”

### Muscles at the posterior side of the thighs

Four muscles, which originated from the ischial tuberosity and were supplied by branches of the sacral plexus, were observed in the posterior aspect of the thighs. The three muscles formed a conjoint tendon and were inserted onto the antero-medial surface of the proximal tibia (Figs 1–3). The attachments and nerve innervations indicated that they were hamstring muscles.

#### 1. *M*. *biceps femoris* (BF)

The origin of this muscle was tendinous, from the ischial tuberosity. BF had a single head, without the short head attaching to the femur. The muscle belly spread out like an isosceles triangle and became aponeurotic. This aponeurosis attached to the superior anterior aspect of tibia (i.e., from the proximal end to the tibial tuberosity) and continued to join with the conjoint tendon. Between this muscle and ST (described later), a muscle bundle connecting these two muscles was found and referred to as the “hamstring bundle” (HB) (Figs 2 and 3). Several branches of the posterior femoral cutaneous nerve descended onto the surface of this muscle. In addition, other cutaneous nerves passed through the muscle belly and innervated the disto-lateral area of the posterior thigh. The BF muscle was supplied by some branches of the DTN, as were the ST and SM (described later) (Fig 3)

#### 2. *M*. *semitendinosus* (ST)

This muscle originated from the ischial tuberosity, became tendinous at the distal part, and then joined the tendon of the gracilis muscle to form a part of the medial conjoint tendon. At the middle of the muscle belly, semitendinosus raphe (STr) was observed. A muscle bundle named the HB in this study, connecting the ST and BF, diverged from this muscle. Both the ST and HB were supplied by the DTN, branches of the sacral plexus. The supplying nerve entered the muscle from the deep surface.

#### 3. *M*. *hamstring bundle* (HB): A muscle bundle between ST and BF

This muscle connecting ST and BF looked similar to the tensor fasciae suralis muscle in humans [36]. However, the directions of the muscle fibers are opposite; the fibers ran from ST to BF in koala (Figs 2 and 3), whereas those in the tensor fasciae suralis are reported to run from BF to ST. The nerve innervating HB was a branch of the DTN, which is a branch from the sciatic nerve, and this muscle is therefore a hamstring muscle. This muscle bundle was observed in six of the eight side sections of the koalas.

#### 4. *M*. *semimembranosus* (SM)

This muscle was located behind the ST muscle, and its origin was the ischial tuberosity. The tendon of this muscle was inserted onto the medial condyle of the tibia. Several nerve branches from the DTN entered the superficial surface of the middle part of the muscle belly (Fig 3).

## Discussion

This study provided two important results for understanding the gross anatomy of koalas. First, by focusing on the innervation as well as the attachment sites of the muscles, it became possible to identify the musculature better than in previous studies. In this way, we succeeded in organizing and updating the findings on muscle morphology in koalas, which had been inconsistent among previous studies. In addition, a muscle identification based on neuro-muscular specificity has enabled us to establish comparison with the eutherians, which has been considered difficult [28]. More numerous studies have been performed to clarify their gross anatomy among eutherian species [12–25, 33, 35, 36]. As previously mentioned, comparison of anatomical findings in marsupials with those in eutherians would be an important insight into understanding the convergent evolution of mammals. This study is the first step in furthering the study of marsupials by enriching the anatomical knowledge that is the foundation of comparative anatomy.

### Updated gross anatomy in the gluteal and posterior thigh regions of koalas

As stated above, the descriptions regarding several muscles were not consistent among previous studies. In addition, many of them were not accompanied by photographs or diagrams, so they could not be verified.

Inconsistencies in descriptions of muscles were particularly pronounced in the gluteal region, where multiple muscles overlap. While there was general agreement on the description of the GSu at the most superficial layer, there were differences in description at deeper levels: especially for the GM muscle, there was disagreement. As is mentioned in the introduction of this study, Macalister [29] and Young [26] have reported that GM of koalas were bilaminar, while Sonntag [30] had not observed the laminations. Our observations in this study supported the former observation, as we confirmed the layered structure of GM muscle in all six koalas. In addition to the koalas in this study, a case in which the GM is clearly divided into two layers has been reported in marsupial wolves [37] and a GM with two portions (not layers) has been reported in tree-kangaroos (*Dendrolagus lumholtzi*) [32].

As for the GMi, we observed it as an undivided single muscle. Similar to the description by Young [26], we found that the positional relationship between the PI muscle and the superior, inferior, and sciatic nerves was unique. Young [26] has described this muscle in koalas as “developed” and its attachments “as usual.” The attachment and innervation suggest that this muscle is the PI, but the three major nerves (superior and inferior gluteal nerves and the sciatic nerves) all emerged superior to the PI, i.e., the suprapiriform foramen, in the koala (Figs 2 and 3). Only one study has reported that the sciatic nerve exits from the suprapiriform foramen in a monotreme species, echidna (*Tachyglossus aculeatus*) [38]. However, generally in mammals, the sciatic nerve is known to emerge from the infrapiriform foramen [13, 14, 39–41], and no similar case has been reported both in eutherians and marsupials. The positional relationship between the PI muscle, superior and inferior gluteal nerves, and sciatic nerve in koalas is unique among mammals.

IF was also found to be inconsistent with the description of previous studies on koalas. In previous studies on marsupial anatomy, muscles with similar attachment sites are sometimes given multiple names that differ between species, leading to confusion (quadrutus femoris [26]; caudo-femoralis [27]; ischiofemoralis [28]). The attachment site and location of the IF observed in this study are consistent with those described by Young [26] as the quadratus femoris muscle, but the attachments and innervation are different from those of the quadratus femoris in eutherian mammals, including humans. First, the muscle observed in koalas is attached to both the sacrum and sacrotuberous ligament, and its origin is located more medially. The insertion was broader and located more distal to the trochanteric fossa. Additionally, the muscle of koalas had only a branch that entered from the posterior (superficial) surface of the muscle belly and did not have a branch that entered from the anterior (deep) surface as does the quadratus femoris muscle of many eutherian mammals, including humans. Finally, this muscle was located in a different layer (more superficial) than the superior and inferior gemellus and the obturator internus muscles. Considering the origin, insertion, innervation, and laminae, we concluded that this muscle is not identical to the so-called quadratus femoris. Based on the site of attachment, it is appropriate to call it the ischiofemoralis muscle.

As for the muscles of the posterior thigh, the types of muscles present and their mutual positional relationships were similar to those of previous studies and other eutherian mammals. The following two points are the exceptions. First, in a previous study, Sonntag [30] described that the BF and SM of koalas arise from a common tendon. Instead, we found that both muscles arise from ischial tuberosity, but from independent origins and not a common tendon (Fig 2). Next, we observed the presence of the HB (a muscle bundle connecting ST and BF) in six of the eight side sections of the koalas we observed. Since it was not observed in all sides of all samples, we cannot rule out the possibility that the presence of this muscle bundle may be a kind of variant. From the nerve innervation and the location, it is certain that this is also part of the hamstrings, but since it is a thin muscle bundle, it does not seem to have powerful function. However, it has not been described in any previous studies.

Thus, in each of the gluteal and posterior thigh regions, we succeeded in clearing up several points of confusion from descriptions in previous studies. Moreover, the identification method based on innervation allows us to compare the function of the same type of muscle with that of eutherians, as described below.

### Comparison of the gluteal and posterior thigh muscles of koalas and other mammals

At present, as mentioned earlier, no study has applied the same muscle identification and description technique as the present study for reporting lower limbs of marsupials. We believe that the widespread use of this muscle-identification method may update classical findings; we have compared the muscle morphology of koalas with that of arboreal primates and other marsupials to the extent possible at this time.

As for the gluteal muscles, the morphology of GSu is different from other taxa. According to Stern [42], various primate species possess GSu with iliac origins, and so do other marsupial species [31, 32]. An iliac origin allows the muscle to perform functions of hip extension and external rotation, but we clarified that koalas’ GSu do not possess this property. The GSu of koalas without iliac origin clearly suggests that the function of this muscle is different. This suggests that the GSu in koalas act mainly as a hip abductor and pulls the femur laterally and dorsally, rather than a hip extensor. However, the lower part of the GSu is expected to have an extensor function due to its similarity with the IF, described below, in terms of the running of muscle fibers. It is known that there is great variation in morphology among species in both placentals and marsupials, even for gluteus medius and gluteus minimus, which are located deeper than the GSu [14, 31, 39–41]. Our study demonstrated that koalas have a two-layered GM (GMS, GMD) and an independent GMi. GMS, which runs mediosuperiorly to lateroanteriorly on the dorsal side of the hip joint and inserts at the lateral side of the greater trochanter to cover the greater trochanter, is assumed to have an abduction function to pull the femur laterally and dorsally. In addition, the caudal part of the GMi (around two-thirds of the total muscle), which runs medially to laterally on the dorsal side of the hip joint and inserts at the great trochanter at the lateral side of the hip joint, is also likely to have an abduction function. Another major function of these gluteal muscles is expected to be hip extension. The GMD passes through the head of the hip joint and inserts at the great trochanter, indicating that it has an extensor function to pull the great trochanter towards the caudal side. In contrast, the cranial side of the GMi may play a role in flexion, which is antagonistic to its extension function; the cranial side of the GMi (approximately one-third of the total muscle) passes through the cranial side of the hip joint and attaches from the base of the great trochanter to the proximal lateral side of the diaphysis, where it pulls the femur cranially and medially, possibly performing flexion and internal rotation.

We consider the possibility that the morphology of GM and GMi might be related to arboreality. Elftman [31] has reported that koalas have a more developed GMi than other marsupials because this muscle works against gravity during arboreal locomotion. It is also reported that arboreal tree kangaroo possessed a developed GM with two portions [32]. Most of the arboreal primate species possesses developed and independent GM and GMi [39–41], and it has been reported that these two muscles are fused in some terrestrial marsupials. For example, the wombat (*Vombatus ursinus*) (considered to be relatively closely related to the koala), the Tasmanian devil (*Sarcophilus harrisii*) [29], and the marsupial mole (*Notoryctes sp*.) have been reported to present with a fusion of the GM and GMi muscles [27], and Sonntag [30] noted that fusion of the muscles was observed in some marsupial species (species unknown).　Conceivably, in terrestrial species, having one fused large muscle with a small range of motion may be more adaptive to the powerful back-and-forth movement of the hind limbs, which enhances propulsion. In contrast, arboreal marsupials, such as the koala, may have been able to ascend and descend tree trunks of various thicknesses by increasing their range of motion through hip abduction and extension. These observations support the inference that each muscle exists independently.

That the IF muscle is present instead of *m*. *quadrutus femoris* is also characteristic of koalas. The quadratus femoris muscle in many mammals including humans acts as an external rotator muscle. However, the ischiofemoralis muscle of koalas is expected to act as a hip extensor rather than an external rotator, considering its attachment site and relationship with the hip joint. Since the IF travels along the dorsal and caudal sides of the hip joint from origin to insertion and reaches the femur, it is likely to pull the femur in the dorsal and caudal directions (hip extension). In addition, the lower part of this IF muscle inserts similar to that of the adductor magnus muscle. Therefore, it is conceivable that it may also have a partial adductor function to prevent over-abduction of the hip joint.

As for the posterior thigh muscles, we observed innervations, attachments, and relative location of the muscles and concluded that SM and ST in koalas would work as hamstring similar to that of other marsupials and arboreal primates. We also found a unique feature of the koala that the pes anserinus is not formed due to the absence of the patella, and the conjugated tendon inserts broadly at the tibia. This is most likely due to the semi-flexed attitude of koalas, as mentioned by Young [26].

Previous studies have discussed the morphology of BF in relation to locomotion style. For arboreal primates, Haxton [43] and Kumakura [44, 45] have proposed that BF morphology corresponds to the varieties of habitual modes of the locomotion: the species which keeps their trunk vertical (vertical climbers or bipedal walkers such as apes and spider monkeys) possess bicipital BF, while horizontal quadrupeds possess monocipital BF. In this study, we observed that BF in koalas is monocipital. As for the other marsupial species, Elftman [31] previously reported that marsupial BF uniformly lacks the short head as found in humans. However, Warburton [32] reported that several Macropodinae species possess bicipital BF. Kangaroos and wallabies show bipedal locomotion with their trunk being vertical. Thus, the correlation between BF morphology and locomotion might be also applicable in marsupials. However, koalas also had a monocipital BF, even though they keep their trunk upright in the tree; they may have preserved the ancestral traits of marsupials.

Although only a part of muscles was described in this study, a similar approach could be continued for other parts of the koala and for other marsupials to allow for a comprehensive and solid discussion of locomotion patterns and muscle morphology in comparison with other phyla. This approach will be useful in understanding adaptive radiation in marsupials.

## Conclusions

In the anatomical studies of marsupials reported to date, muscle identification has been based exclusively on attachment sites. Therefore, comparison with other phylogenetic groups is challenging due to the lack of homology. In the present study, we updated the knowledge of koala muscles and resolved the discrepancies that existed among the previous findings by identifying muscles based on their innervation. This muscle identification method focuses on the ontogenetic origin of the muscle.

Moreover, we have established a standard for the comparison of marsupial and eutherian anatomy. The utility and value of muscle identification based on innervation is that it allows for corresponding features in different species to be identified, which allows us to better infer muscle morphology and function and to depict sundry characteristic musculoskeletal morphologies. However, this study was limited in its scope because we also wished to demonstrate the utility and value of this technique of muscle identification. To further analyze the anatomical features involved in koala locomotion, we hope to apply these techniques to the other muscles of the thigh as well as the erector spinae muscle group. Future analysis of koala anatomy and comparison to the corresponding features in eutherians will illustrate a comprehensive picture of the features that distinguish the koala from other species.
